# Sexual dimorphic miRNA-mediated response of bovine elongated embryos to the maternal microenvironment

**DOI:** 10.1371/journal.pone.0298835

**Published:** 2024-02-29

**Authors:** Dessie Salilew-Wondim, Michael Hoelker, Eva Held-Hoelker, Franca Rings, Ernst Tholen, Christine Große-Brinkhaus, Karl Shellander, Carina Blaschka, Urban Besenfelder, Vita Havlicek, Dawit Tesfaye

**Affiliations:** 1 Department of Animal Science, Biotechnology and Reproduction of Farm Animals, University of Göttingen, Göttingen, Germany; 2 Institute of Animal Sciences, Animal Breeding, University of Bonn, Bonn, Germany; 3 Institute of Animal Breeding and Genetics, University of Veterinary Medicine Vienna, Vienna, Austria; 4 Department of Biomedical Sciences, Animal Reproduction and Biotechnology Laboratory, Colorado State University, Fort Collins, CO, United States of America; University of Edinburgh, UNITED KINGDOM

## Abstract

A skewed male-to-female ratio in cattle is believed to be due to the biased embryo losses during pregnancy. The changes in biochemical secretion such as miRNAs by the embryo due to altered maternal environment could cause a sex biased selective implantation resulting in a skewed male to female ratio at birth. Nevertheless, it is still not clear whether the male and female embryos could modify their miRNA expression patterns differently in response to altered physiological developmental conditions. Therefore, this study was focused on identifying sex specific miRNA expression patterns induced in the embryo during the elongation period in response to the maternal environment. For this, in vitro produced day female and male embryos were transferred to Holsteins Frisian cows and heifers. The elongated female and male embryos were then recovered at day 13 of the gestation period. Total RNA including the miRNAs was isolated from each group of elongated embryo samples were subjected to the next generation miRNA sequencing. Sequence alignment, identification and quantification of miRNAs were done using the miRDeep2 software package and differential miRNA expression analyses were performed using the edgeR bioconductor package. The recovery rate of viable elongating embryos at day 13 of the gestation period was 26.6%. In cows, 2.8 more viable elongating male embryos were recovered than female embryos, while in heifers the sex ratio of the recovered elongating embryos was close to one (1.05). The miRNA analysis showed that 254 miRNAs were detected in both male and female elongated embryos developed either in cows or heifers, of which 14 miRNAs including bta-miR-10b, bta-miR-148a, bta-miR-26a, and bta-miR-30d were highly expressed. Moreover, the expression level of 32 miRNAs including bta-let-7c, bta-let-7b, bta-let-7g, bta-let-7d and bta-let-7e was significantly different between the male and female embryos developed in cows, but the expression level of only 4 miRNAs (bta-miR-10, bta-mR-100, bta-miR-155 and bta-miR-6119-5p) was different between the male and female embryos that were developed in heifers. Furthermore, 19 miRNAs including those involved in cellular energy homeostasis pathways were differentially expressed between the male embryos developed in cows and heifers, but no significantly differentially expressed miRNAs were detected between the female embryos of cows and heifers. Thus, this study revealed that the sex ratio skewed towards males in embryos developed in cows was accompanied by increased embryonic sexual dimorphic miRNA expression divergence in embryos developed in cows compared to those developed in heifers. Moreover, male embryos are more sensitive to respond to the maternal reproductive microenvironment by modulating their miRNA expression.

## Introduction

Several lines of evidence have shown a skewed male-to-female ratio in cattle [1–6], and other mammals including humans [7] at birth. This could be associated with sex biased embryo losses during development. In addition, at early phase of the lactation, high yielding dairy cows usually suffered from metabolic stress due to occurrence of negative energy balance lactation [8]. As the result of this, embryo losses is more frequent in cows than heifer [9].

Although deciphering major factors contributing to the skewed male-to-female ratio remains the subject of subsequent investigation, several factors including time of insemination [4], maternal condition [10], speed of development and morphology of the embryos [11–14], stages of embryonic development before transfer [15], embryo manipulation technique such as intracytoplasmic sperm injection (ICSI) or in vitro fertilization (IVF) [16] are believed to contribute to the altered male to female ratio at birth.

Among the factors listed above, the pre-conception maternal condition which was proposed by Trivers and Willard [10] is more relevant when the recipient’s physiological condition induces embryo losses in mammals. For instance, in Barbary sheep [17], dairy cattle [18] and pigs [19] while better maternal body condition favors birth of more males, the poor maternal body conditions favors female newborns. Under in vitro culture conditions, high glucose concentration in the culture media seems to favor the development of male embryos compared to female ones [20]. In addition, the biochemical contents (mRNAs and proteins) of embryos, and the physical and biochemical milieu of the maternal tract cause sex-biased selective implantations and skewed male-to-female ratio at birth [see review [21]]. For instance, in rats, in-utero insults during the peri-conception period may be risky for female embryo viability resulting in more males produced at birth while in-utero insults during mid- to late-gestation could risk male embryos resulting in more females surviving until birth [22]. This can be related to sex specific biochemical alteration in embryo and/or maternal environment. For instance, under in vitro conditions, the female bovine embryos can produce double the amount of pregnancy signalling factor, the IFN-tau, than the amount produced by male embryos [20, 23]. Gene-expression analysis between male and female blastocysts by DNA microarray also indicated the upregulation of genes associated with signal transduction and cell differentiation and downregulation of genes associated with metabolic process and cell cycle in female blastocysts compared to the male ones [24]. This altered gene expression is believed to lead to sex-specific embryonic development or sex-specific embryonic losses [25]. A study by Heras et al. [26] showed differential expression of 119, 54 and 48 genes between the male and female derived from vivo, cultured in serum-containing and cultured in serum-free medium, respectively. These and other similar findings may suggest that the embryo of one sex may signal its presence in the maternal environment more robustly than the other one by secreting mRNA signals. Thus, further investigation is required to understand the gene regulatory mechanisms such as microRNAs (miRNAs) that are responsible for the survival of one sex than the other one under different physiological developmental conditions.

MicroRNA, small non coding RNAs, are known to be one of the posttranscriptional regulators of gene expression [27]. These tiny non coding RNAs were detected at various embryonic stages and are implicated in embryonic development and implantation (see review, [28]. For instance, differential expression miRNAs between the 4- and 8-cell stages [29], unhatched and hatched bovine blastocysts [30], inner cell mass and trophectoderm [31], embryonic outgrowth and the blastocyst stage embryos [32] could indicate the stage specific role of miRNAs during preimplantation development, cell differentiation and implantation.

Interestingly, the uterus secretes specific miRNAs which may be taken up by the embryo to modify the transcriptome profiles to facilitate implantation [33]. Conversely, it is also suggested that miRNAs secreted by embryos can be taken by endometrial epithelial cells, and cause a change in the endometrial function [34]. Previous studies also demonstrated that the female and male bovine blastocysts secret miRNAs into the culture media which can be taken up by the primary bovine endometrial epithelial [35]. This may provide a clue that male and female embryos may signal to the maternal environment differently by releasing male and female specific miRNAs. However, several questions associated with sex specific adaptations of embryos need further investigation with respect to the in vivo embryogenesis and embryo losses during embryo elongation, which is the critical stage that coincides with significant embryo losses in cattle [36]. In addition, since high yielding dairy cows are affected by metabolic stress induced by negative energy balance, pregnancy failure is more common in cows than heifers [9]. Nevertheless, the question, of whether male and female embryos respond differently to the maternal microenvironments via altered miRNA expression profiles during the elongation stage, remains unanswered. Although male and female embryos showed intrinsic differences in various epigenetic modifiers, including small noncoding RNAs, whether those conceptuses respond to maternal microenvironment via changing their miRNA expression pattern is not yet known. Thus, the current study aimed to investigate the miRNA-expression profiles and associated molecular pathways in elongating male and female bovine embryos developed in cows and heifers. The results from this study will contribute basic data for the identification of miRNAs that are indicators of the sexual dimorphic response of embryos to the maternal microenvironment during the critical periods of embryogenesis in mammals in general and bovine species in particular.

## Materials and methods

### Animal handling and management

All experimental animals (cows and heifers) used for this study received similar total mixed rations, and they were kept in the same farm and housing conditions. Handling and management of experimental animals have adhered to the rules and regulations of the German law of animal protection. The experiment dealing with animals was approved by the Animal Welfare Committee of the University of Bonn with proposition number 84–02.05.20.12.075.

### In vitro maturation, in vitro fertilization and in vitro culture

**Fig 1:**
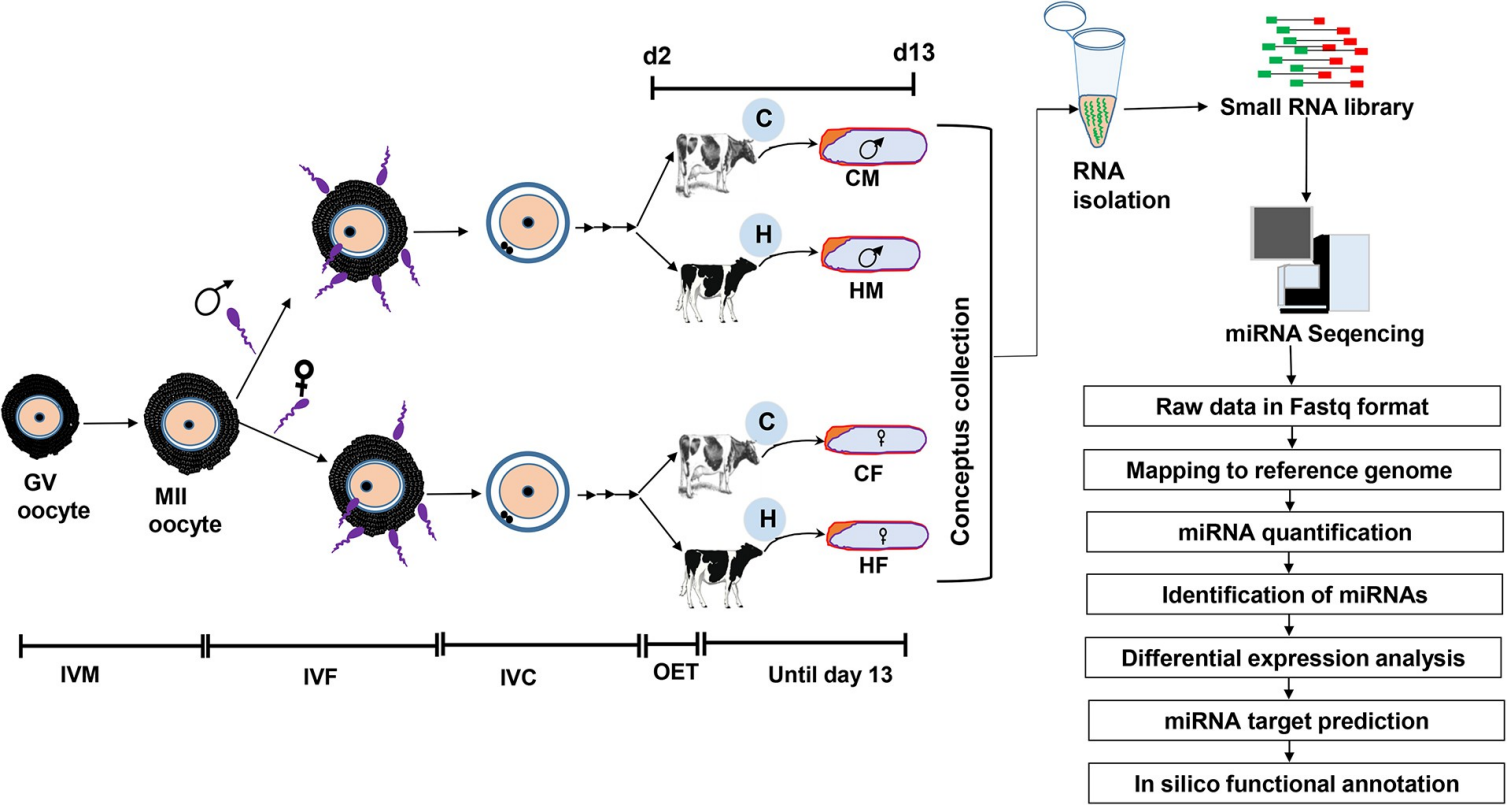
The experimental outline indicating the generation of in vitro day 2 sexed embryos and transfer to cows (C) and heifers (H), recovery of four groups of day 13 embryos (CM, CF, HM & HF) and data analysis steps. IVM: in vitro maturation, IVC: In vitro culture, OET: Oviductal embryo transfer.

In the current study, we used in vitro fertilization using sex-sorted semen to produce day 2—male and female embryos for nonsurgical endoscopic oviductal transfer into Holstein Friesian cows with parity two and heifers. Therefore, this study involved in vitro oocyte maturation, in vitro fertilization (using sex-sorted semen), in vitro culture and endoscopic-guided transfer of preimplantation stage embryos into the oviduct. The overview of the experimental design is indicated in Fig 1. Cumulus oocyte complexes (COCs) were collected from the ovaries of slaughterhouses and in vitro matured using a similar protocol used in our previous study [37]. At the end of in vitro maturation, a group of 50 COCs were in vitro fertilized with either X or Y-chromosome-bearing sperm derived from Holstein Frisian bull. For this, frozen thawed sex sorted sperm were subjected to a swim-up procedure. In vitro fertilization (2 × 10 ^6^ sperm in fertilization droplet) was performed in Fert-TALP medium supplemented with 20 μM penicillamine, 10 μl PHE (Hypotaurine-Epinephrin-solution), 6 mg ml^−1^ BSA-FFA, 50 μg ml^−1^ gentamycin, and 1 μg ml^−1^ heparin. The COCs and sperm were co-incubated for 18 h. After the removal of cumulus and sperm cells and washings, the two zygote groups were in vitro cultured in 400 μl of synthetic oviductal fluid (SOF) culture medium supplemented with 0.6% fatty acid-free bovine serum albumin (BSA) under 5% O_2_, 5% CO_2_ and 39°C until day 2 prior transferring to recipients.

### Oviductal transfer of male or female embryos to cows and heifers

Transfer of embryos on day 2 and recovery on day 13 of the gestation period was required to get an overview of the effect of the maternal environment (oviduct and the uterine challenges) until embryo elongation. To perform this, Holstein Frisian heifers (n = 11) between 15–20 months of age and with no history of calving and Holstein Frisian cows (n = 10) with parity two were oestrous synchronized by intra-muscular administration of 500 mg of the prostaglandin F2α (PGF2α) analogue cloprostenol (Estrumate; Munich, Germany) twice within 11 days interval. Each of the PGF2α treatments was followed by the administration of 0.02 mg GnRH-analogue buserelin (Receptal) (Intervet, Boxmeer, the Netherlands). Afterwards, 6 cows and 6 heifers each received day 2 female embryos (n = 20 per recipient), while the other 4 cows and 5 heifers received day 2 male embryos (n = 20 per recipient) using endoscopic embryo tubal transfer [38].

### Day 13 elongated embryo recovery

On day 13 of the gestation period, elongated embryos were recovered after the targeted slaughter of the pregnant animals from the nearby slaughterhouse. All experimental animals were slaughtered and male and female elongated embryos were recovered. The elongated embryos were then classified according to their sex and origin. Male and female elongated embryos derived from cows were named CM and CF, respectively and male and female elongated embryos derived from heifers were named HM and HF, respectively. All samples (CM, CF, HM and HF) were initially snap-frozen in liquid nitrogen and stored at -80°C until further analysis.

### Total RNA isolation

Total RNA including miRNA was isolated individually from 84 samples (CM = 20, CF = 13, HM = 24, HF = 27) using AllPrep DNA/RNA/miRNA Universal Kit following the manufacturer’s protocol. Briefly, each of the elongated embryos was repeatedly vortexed in 300 μl lysis buffer containing β-mercaptoethanol (1%), transferred into QIAshredder and centrifuged for 2 minutes at maximum speed. The supernatant was transferred into the DNA spin column and centrifuged at full speed for 30 seconds to trap the DNA and allow the RNA to pass. Afterwards, 50 μl Proteinase K and 200 μl 100% ethanol were added and incubated at room temperature for 10 min. At the end of the incubation period, 400 μl of 100% ethanol was added and the sample was transferred into RNeasy® spin column placed in a 2 ml collection tube and centrifuged for 15 s. Washing of the sample was performed by centrifugation of the sample in 500 μl Buffer RPE for 15 s. DNA contamination was eliminated by performing on-column DNA digestion. After successive washings in 500 μl Buffer RPE and 500 μl of 96–100% ethanol, total RNA was recovered in 35 μl RNase-free water. The quality and quantity of RNAs were evaluated using Agilent 2100 bioanalyzer integrated with RNA 6000 Nano LabChip® Kit (Agilent Technologies Inc, CA, USA) and Nanodrop 8000 Spectrophotometer (Thermo Fisher Scientific Inc, DE, USA), respectively. A total of 20 individual RNA samples (5 RNA samples for each CM, CF, HM and HF group) with RNA integrity number (RIN) ≥ 6, A260/A280 = 1.8–2.2 and with a total concentration of ≥ 500 ng were selected for the next generation miRNA sequencing.

### Small RNA library preparation and sequencing

Small RNA library preparations and miRNA sequencing were done by GENEWIZ Germany GmbH (currently, GENEWIZ from Azenta Life Sciences). Briefly, small RNA libraries were performed using TruSeq small RNA library preparation (Illumina, San Diego, CA) from a total of 20 samples (5 RNA samples as biological replicates for each experimental group). Illumina 3’ and 5’ adapters were ligated to the RNA molecules with a 5’-phosphate and a 3’-hydroxyl group sequentially using ≥ 500 ng total RNA as input. This was followed by reverse transcription. Accordingly, cDNA constructs were enriched by PCR amplification using primers that annealed to the adapter ends. The amplified cDNA construct was purified by polyacrylamide gel electrophoresis, and the correct band (~145–160 bp) was excised from the gel and eluted with water. The eluted cDNA was concentrated by ethanol precipitation. The libraries for sequencing were validated on the Agilent TapeStation 4200 (Agilent Technologies, Palo Alto, CA, USA), and quantified using Qubit 2.0 Fluorometer (Invitrogen, Carlsbad, CA) and quantitative PCR (KAPA Biosystems, Wilmington, MA, USA). The sequencing libraries were clustered on one flow cell. After clustering, the flow cell was loaded on the Illumina instrument (4000 or equivalent) according to the manufacturer’s instructions. Image analysis and base calling were conducted by the control software. Raw sequence data (.bcl files) generated by the sequencer were converted into fastq files and de-multiplexed using Illumina’s bcl2fastq 2.17 software. One mismatch was allowed for index sequence identification.

### Raw data quality assessment and adapter trimming

Quality evaluation of the raw sequence data was done with FastQC, a free available sequence analysis tool, (http://www.bioinformatics.babraham.ac.uk/publications.html). The basic statistics, the sequence quality, quality score, sequence content and GC content of each raw sequence were evaluated and raw data that fulfilled basic quality parameters were used for detection and expression analysis. Afterwards, we used miRDeep2 a software package to further process the raw sequence data. Adapters and low quality ends were trimmed using cut adapt and sequences with less than 18 bp were removed from downstream analysis.

### Raw sequence alignment, identification and quantification of miRNAs

Prior to sequence alignment, packages including RNAfold, randfold and the perl packages PDF:API and TTF were installed in the R software environment. The miRBase reference files were downloaded and the mature miRNA and hairpin sequences were extracted using extractmiRNAs.pl and extract_miRNAs.pl, respectively. The bovine reference genome (Genome assembly: ARS-UCD1.2) was indexed using bowtie-build version 1.3.0 https://sourceforge.net/projects/bowtie-bio/files/bowtie/1.3.0/). Following this, the fastq files were parsed into fasta format, sequences with non-canonical letters were discarded, and identical reads were collapsed and mapped to the indexed bovine reference genome using the bowtie tool. Detection of miRNAs were detected by miRDeep2.pl module using collapsed sequencing reads, the bovine reference genome fasta file, mapped reads, known mature, and precursor miRNAs of *Bos taurus* and mature miRNAs from *Homo sapiens* were used as inputs. Quantification of miRNAs was done by mapping sequencing reads to miRNA precursors and subsequently the mature miRNA sequences to the predefined precursors.

### Identification of highly expressed and differentially expressed miRNAs

Prior identification of highly expressed and differentially expressed miRNAs, the CSV file generated by the quantifier module of the miRDeep2 software was imported into the R program. Differential expression analyses were performed using the edgeR bioconductor package [39]. For this, first, the library size of each sample was determined, and the library sizes were normalized by setting a set of scaling factors using a trimmed mean of M-values (TMM) between samples. Quantile-adjusted conditional maximum likelihood common dispersion, trended dispersions and tagwise dispersion were estimated using the estimateDisp function. The mean expression differences between samples were tested with a quasi-likelihood F-test (Robinson et al. 2010) and the false discovery rate method [40] was used to implement multiple hypothesis testing correction. The miRNAs were considered to be significantly differentially expressed between groups when the absolute value of the fold-change (FC) was higher than 1.5 (|FC| > 1.5) and the p-value < 0.05 and false discovery rate (FDR) < 0.1.

### Functional annotation of highly expressed and differentially expressed miRNAs

The functional annotation of highly expressed and differentially miRNAs was determined by using their predicted target genes. Target gene prediction was done by miRNet, a miRNA-centric network visual analytics platform [41] using the bovine and the human orthologue. Afterwards, enriched pathways were identified using g:profiler tool [42]. For this, gene lists associated with miRNAs were imported to the program and *Bos taurus* species was selected on the organisms’ dropdown menu. Pathway enrichment analysis was performed by considering all bovine gene lists available in the Ensembl database and Benjamini-Hochberg p-value correction was used to identify significant pathways (Reactome and KEGG pathways).

## Results

### Embryonic development

In this study, following the transfer of the day 2 embryos, the recovery rate of viable elongating embryos on day 13 of the gestation period was 26.6% and the overall sex ratio was skewed towards males (Table 1). The morphology of the recovered embryos ranges from an ovoid to tubular shape and their size ranges up to 2 mm. The recovery rate of viable elongating embryos in cows and heifers was almost similar, but in cows, 2.8 more viable elongating male embryos than females were obtained. In heifers, the sex ratio of the elongating embryos was 1.05 (Table 1).

**Table 1:** Number of day 2 female and male embryo transferred, the recovery rate and sex ratio of the embryos on day 13 of the gestation period.

Recipient group	Embryo group	Total embryo transferred	Viable elongated embryos recovered (%)	Sex ratio of viable embryos (Male/female)
Cows	Male	80	42.0	2.80
	Female	120	15.0	
	Total	200	26.0	
Heifers				
	Male	100	28.0	1.05
	Female	120	26.6	
	Total	220	27.2	
Overall	Male	180	34.4	1.60
	Female	240	20.8	
	Total	420	26.6	

### MicroRNAs required for embryo elongation

Twenty small RNA libraries (five for each group) were generated in the four sample groups (CM, CF, HM and HF) and 12.9 million reads and 3763.3 Mbases per sample were generated. The mean read quality score was 30.4 and about 43, 28, 36 and 29% of the reads in CM, CF, HM and HF, respectively were mapped to the reference bovine genome.

**Fig 2:**
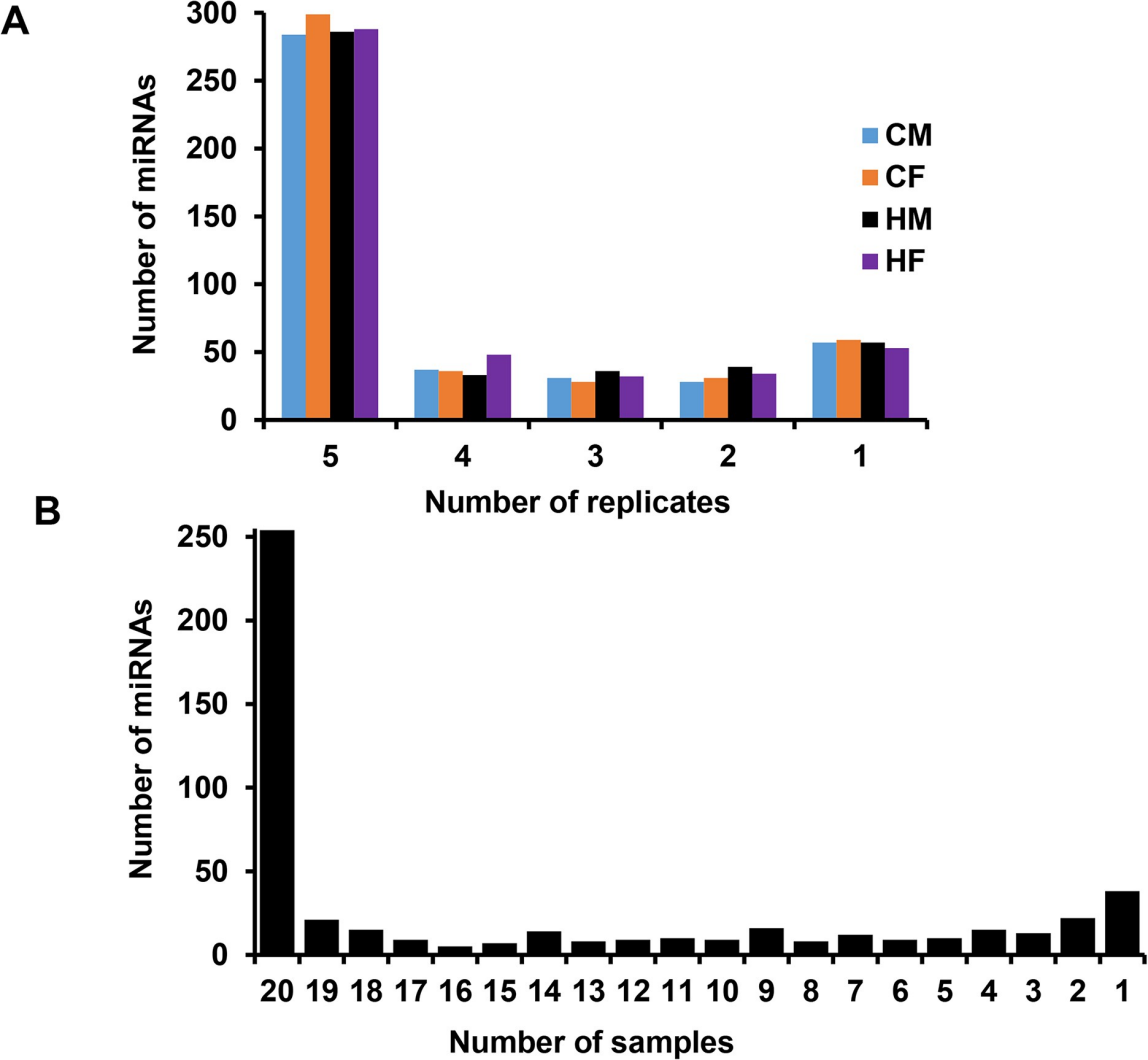
The number of detected miRNAs in male and female embryos developed in cows or heifers at day 13 of gestation. A) The number of known miRNAs detected in 5, or only in 4, 3, 2 or 1 replicate/s (embryo/s) of CM, CF, HM and HF groups. B) The number of known miRNAs detected in 20 or less samples when each replicate was considered as a sample and the groups were merged. A total of 20 samples represent 5 replicates each in CM, CF, HM and HF groups. A total of 254 miRNAs were detected in all 20 samples.

**Fig 3:**
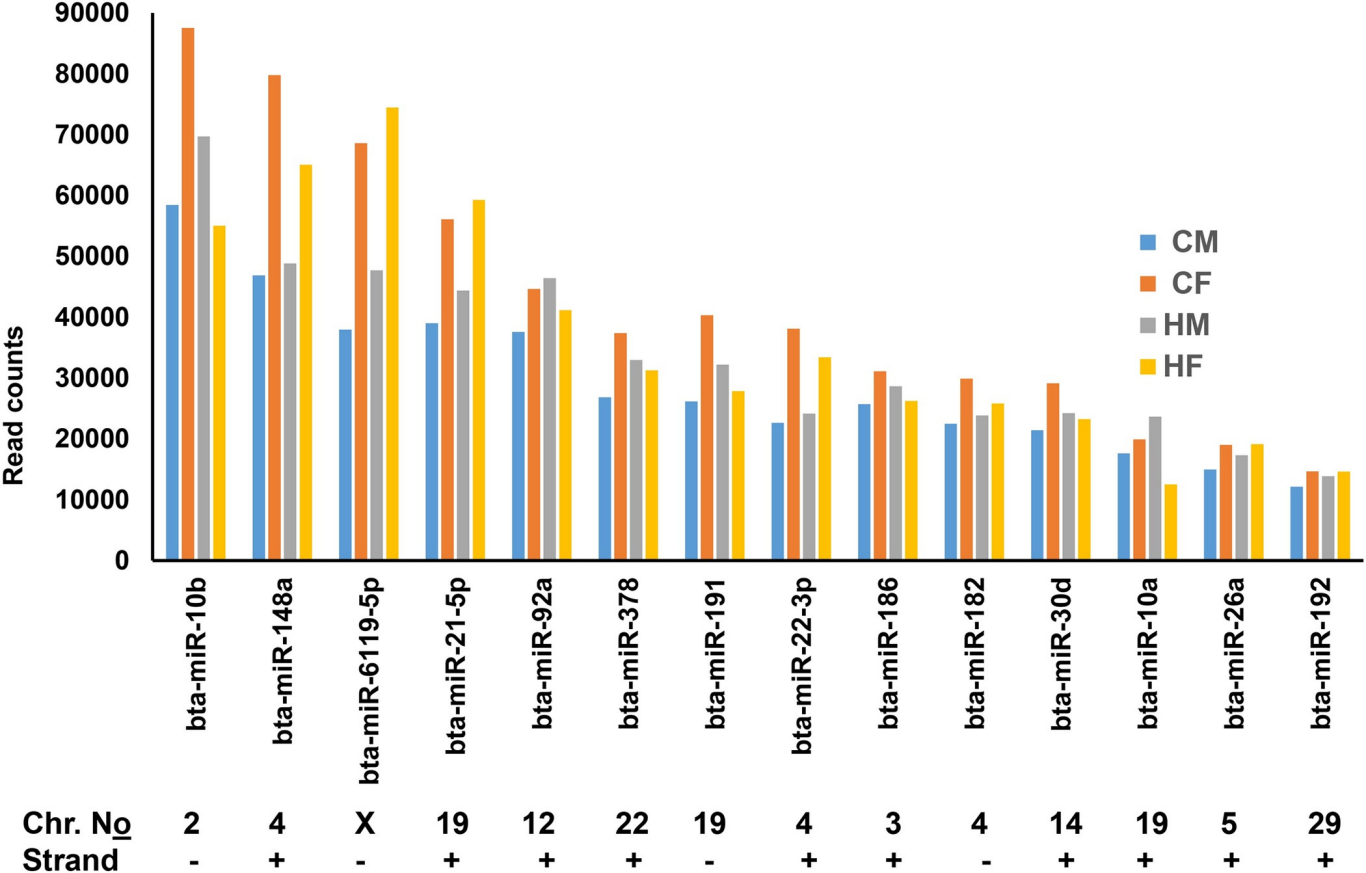
The expression level of the top 14 detected miRNAs in in CM, CF, HM and HF groups.

Prior the identification of differentially expressed miRNAs, we evaluated the miRNAs expressed in each sample group. For this, first, we filtered miRNAs which showed at least 5 read counts in at least three of the five biological replicates. Accordingly, 353, 363, 355 and 368 known bovine miRNAs were detected in the CM, CF, HM and HF samples, respectively. Of these, 284, 299, 286 and 288 miRNAs were detected in all 5 biological replicates of the CM, CF, HM and HF samples, respectively (Fig 2A). When all samples irrespective of the group were considered, a total of 254 miRNAs were commonly detected in all 20 samples (Fig 2B, S1 Table). Of these, 14 miRNAs, bta-miR-10b, bta-miR-148a, bta-miR-6119-5p, bta-miR-21-5p, bta-MiR-92a, bta-miR-378, bta-miR-191, bta-miR-22-3p, bta-miR-186, bta-miR-182, bta-miR-30d, bta-miR-10a, bta-miR-26a, bta-miR-192 were expressed in all samples with average read counts of 12,000–67682 (Fig 3). These miRNAs are localized on chromosomes X, 2, 3, 4, 5, 12, 14, 19, 22 and 29.

**Fig 4:**
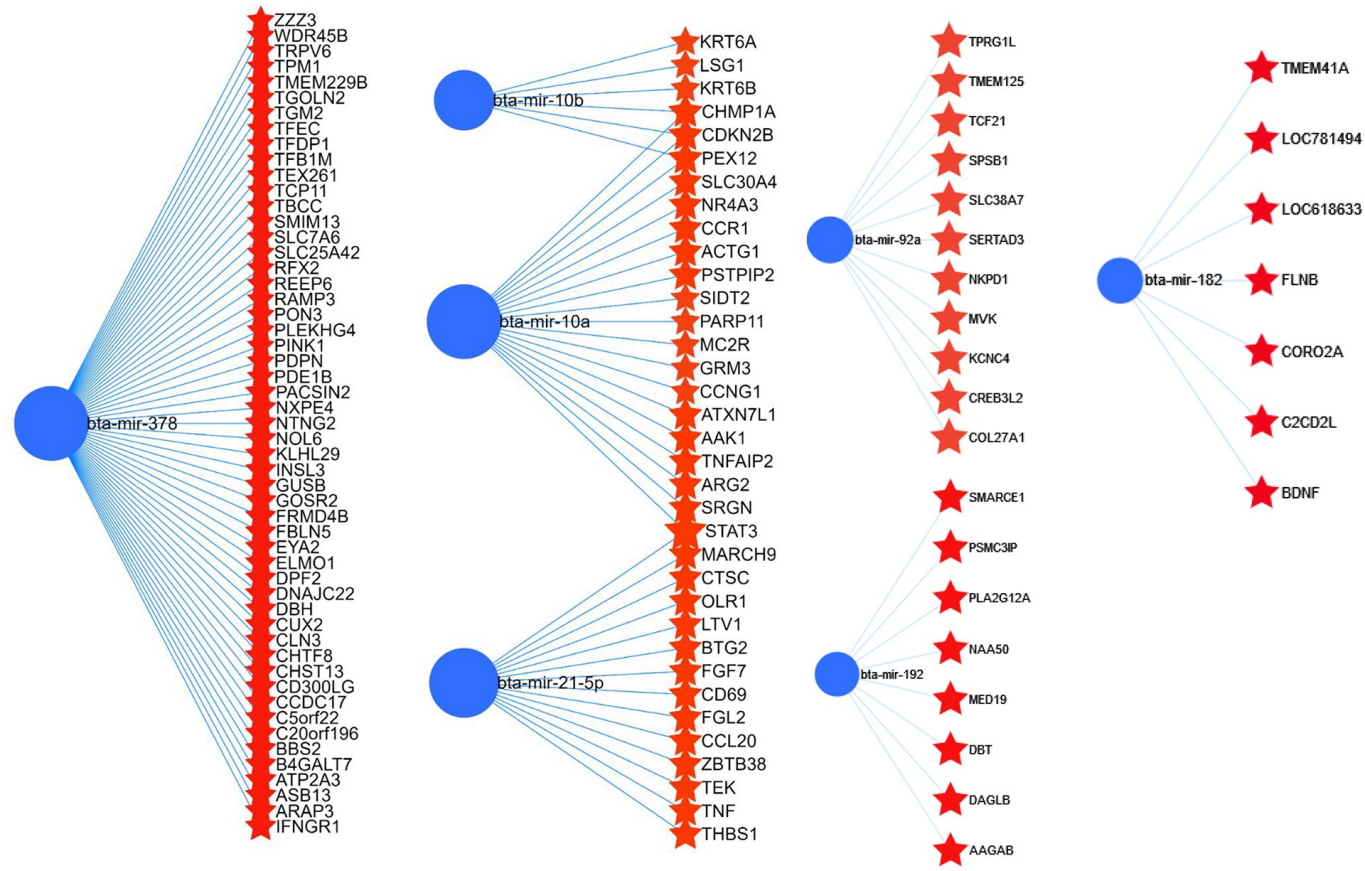
The interaction of highly expressed bovine miRNAs and their target genes.

**Fig 5:**
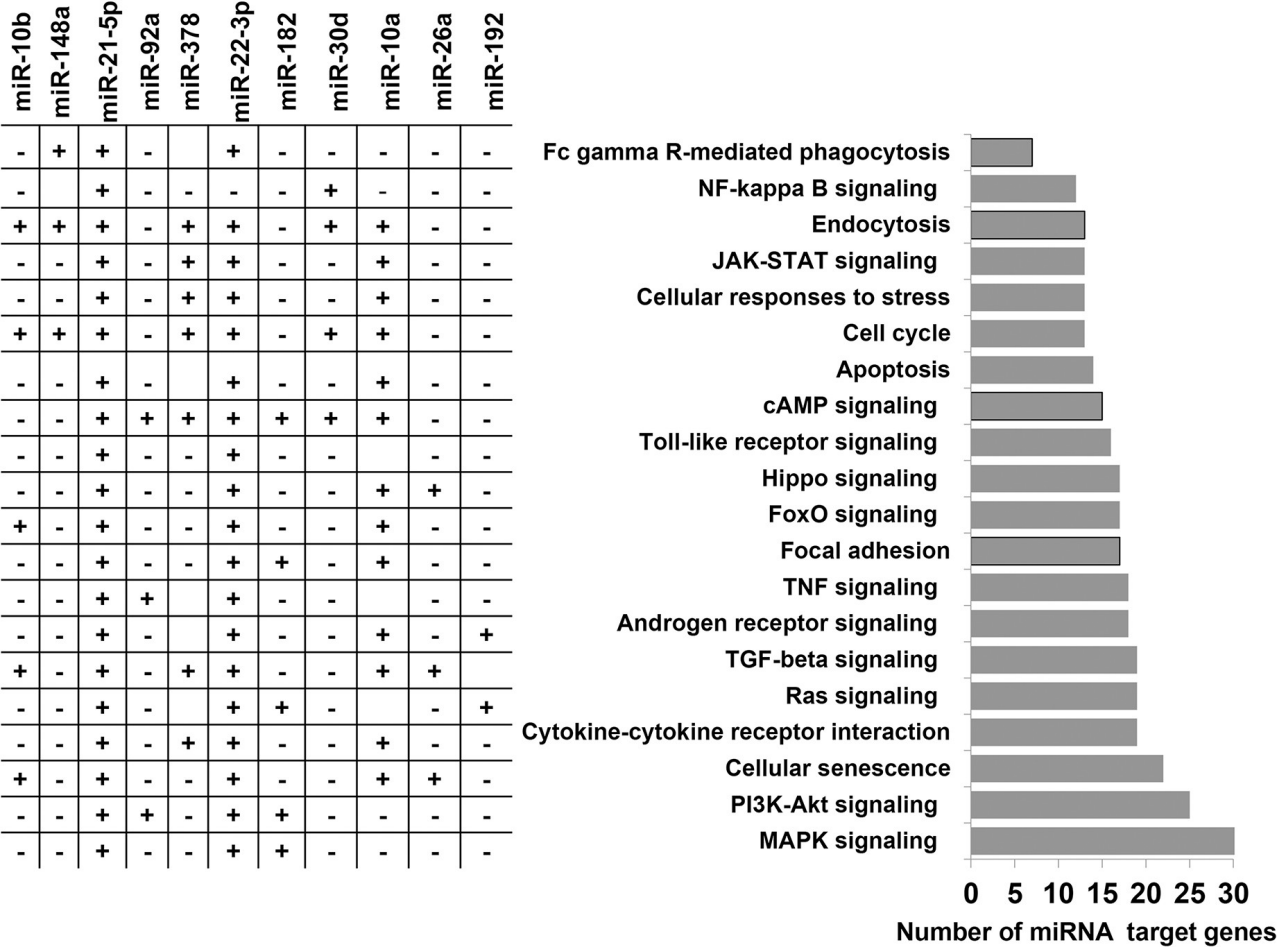
Molecular pathways enriched by highly expressed miRNAs. The plus (+) and minus (-) symbols indicate the presence and absence of the miRNAs in the specific pathway.

To get an overview of the relevance of those highly expressed 14 miRNAs in bovine embryo elongation, an in silico functional characterization was performed based on their predicted bovine genes and/or validated target genes that were obtained from bovine and human databases. Accordingly, among the highly expressed miRNAs, bta-mir-378, bta-mir-10a, bta-mir-10b, bta-mir-182, bta-mir-192, and bta-mir-92a were associated with 433 genes. The representative interaction of miRNAs and their target genes is indicated in Fig 4 and S2 Table. Among the total target genes compiled for downstream analysis, the miRNA-target interaction networks analysis using miRnet software also indicated 103 target genes of miR-21-5p including *SMAD7*, *MARCA4*, *IGF1R*, *SOD3* and *PTX3* and 46 target genes of miR-22-3p including *PTEN*, *CDKN1A*, *BMP7*, *BMP6* and *ADORA2A* have been validated using luciferase assay (S3 Table). Pathway analysis using their validated and predicted target genes indicated that those highly expressed miRNAs were involved in several pathways including immune response related pathways (Toll-like receptor signaling, cytokine-cytokine receptor interaction, TNF signaling, NF-kappa B signaling), cell cycle and apoptosis, cell-to-cell communication related pathways (focal adhesion, endocytosis), Hippo and Foxo signaling pathways (Fig 5).

#### Sexual dimorphic miRNA expression patterns in elongated embryos developed in cows

**Fig 6:**
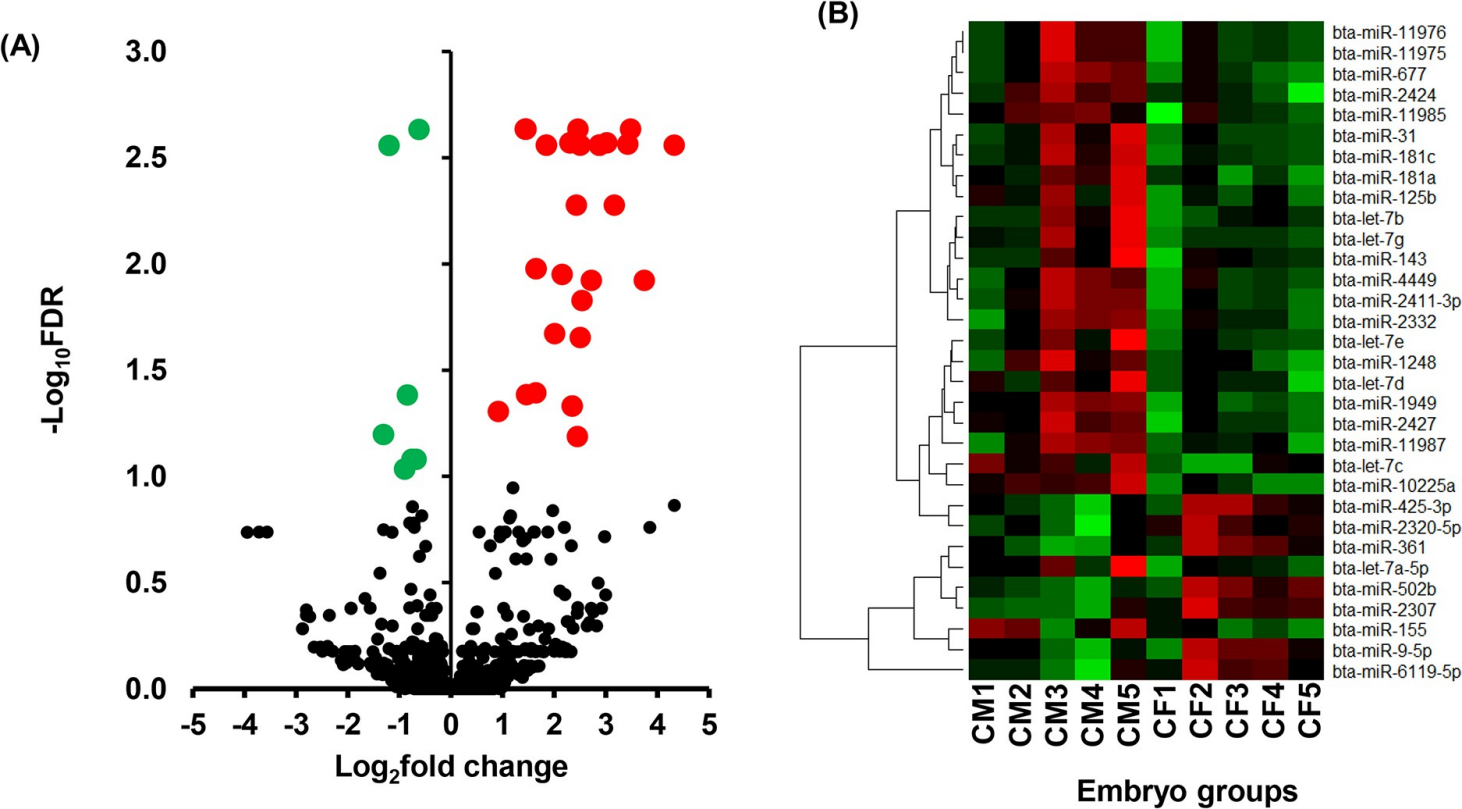
Differentially expressed miRNAs between CM and CF. A) Volcano plot showing the average (log_2_ fold change) expression of miRNAs in CM compared to CF. Red and green dots represent the up and downregulated miRNAs, respectively in CM compared to the CF group. The black dots represent miRNAs that did not show any significant expression differences between CM and CF. B) The heatmap representing the expression patterns of differentially expressed miRNAs within and across the biological replicates. The red and green colors represent increased and decreased miRNA expression.

Following the identification of miRNAs expressed in each sample group, next we identified differentially expressed miRNAs (DEmiRNAs) by comparing their abundances in male and female elongated embryos developed in cows. The results have shown 25 miRNAs were significantly upregulated and 7 other miRNAs were downregulated in males compared to the female elongated embryos (Fig 6). Among those, 11 miRNAs including the let 7 families (bta-let-7c, bta-let-7b, bta-let-7g, bta-let-7d, bta-let-7e) were increased by > 4 folds in males compared to the female elongated embryos (Table 2).

**Table 2:** Top differentially expressed miRNAs between the CM and CF embryo groups.

miRNA	Log_2_ fold change	P value	FDR
bta-miR-11987	4.3	0.0000	0.0028
bta-miR-10225a	3.7	0.0003	0.0120
bta-miR-1949	3.5	0.0000	0.0023
bta-miR-2427	3.4	0.0000	0.0027
bta-miR-11975	3.2	0.0001	0.0053
bta-miR-2411-3p	3.0	0.0000	0.0027
bta-miR-11975	2.9	0.0000	0.0028
bta-miR-11976	2.9	0.0000	0.0028
bta-miR-4449	2.7	0.0003	0.0120
bta-miR-2332	2.5	0.0003	0.0149
bta-miR-11985	2.5	0.0005	0.0223
bta-miR-677	2.5	0.0000	0.0028
bta-miR-181c	2.5	0.0000	0.0023
bta-let-7c	2.5	0.0020	0.0652
bta-let-7b	2.4	0.0001	0.0053
bta-miR-1248	2.4	0.0013	0.0468
bta-let-7g	2.3	0.0000	0.0027
bta-miR-31	2.3	0.0000	0.0027
bta-let-7d	2.2	0.0002	0.0112

FDR: False discovery rate

**Fig 7:**
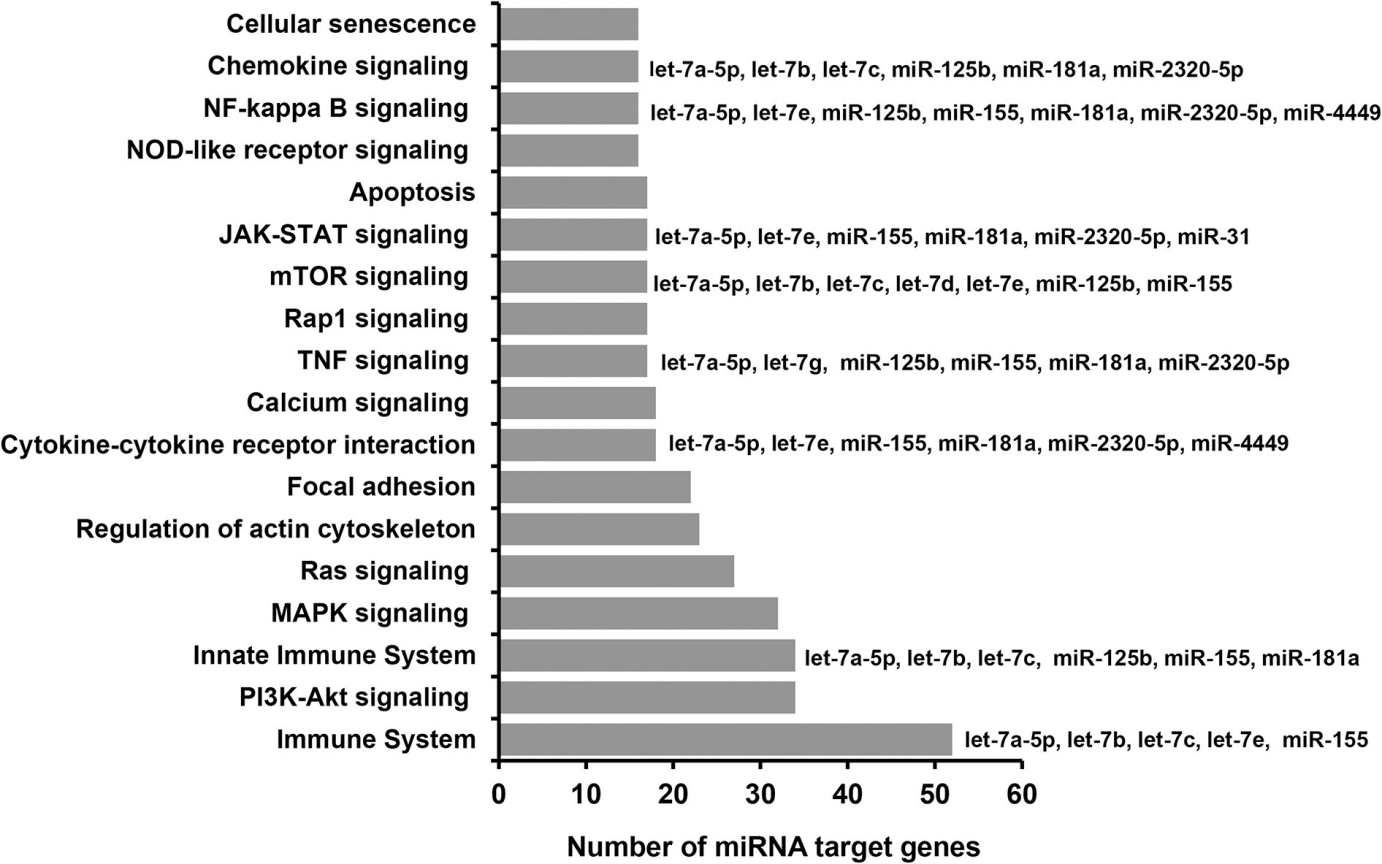
Molecular pathways enriched by differentially expressed miRNAs between CM and CF.

The functional relevance of these DEmiRNAs in embryo development and survival was analysed via their target genes. For this, first, we identified experimentally validated and in silico predicted target genes of these miRNAs. Accordingly, 24 miRNAs including the let 7 families (let-7a-5p, -7b, -7c, -7d, -7e, -7g), bta-miR-181a and bta-miR-181c were predicted to target 1232 genes. Among these, the miRNet identified 43 genes including *AGO1* and *AGO4* as validated target genes of the let-7a-5p miRNA (S4 Table). Pathway analysis using the target genes indicated that these miRNAs are involved in various pathways including the mammalian target of rapamycin (mTOR) signalling, immune response related pathways (innate immune response, NF kappa B signalling, cytokine-cytokine receptor interaction, TNF signalling), focal adhesion, apoptosis (Fig 7, S5 Table). Interestingly, Let-7a-5p and let-7b were involved in many of these pathways including the immune system, innate immune and mTOR signaling pathways.

### Dimorphic expression patterns of miRNAs in elongated embryos developed in heifers

**Fig 8:**
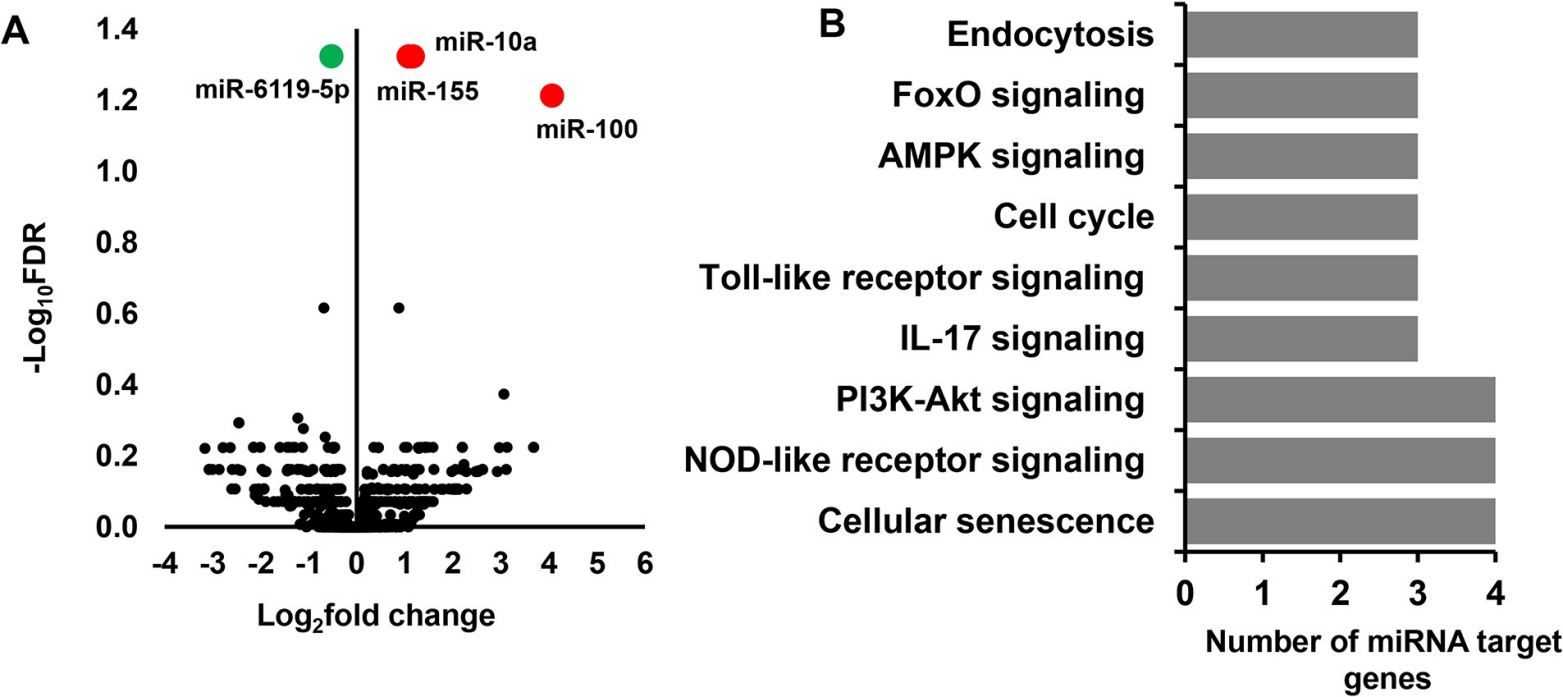
Differentially expressed miRNAs between HM and HF. A) Volcano plot showing the average (log_2_ fold change) expression of miRNAs in HM compared to HF. Red and green dots represent the up and downregulated miRNAs, respectively in HM compared to the HF group. The black dots represent miRNAs that did not show significant expression differences between the HM and HF. B) Molecular pathways enriched by differentially expressed miRNAs between HM and HF.

The DEmiRNAs between the male and female embryos developed in heifers were analyzed using a similar method that was used in male and female embryos developed in cows. Results indicate that only 4 miRNAs (bta-miR-6119-5p, bta-miR-10a, bta-miR-155 and bta-miR-100) were significantly differentially expressed between the male (HM) and female (HF) embryos developed in heifers (Fig 8A). Among these, bta-miR-155 and bta-miR-6119-5p were also differentially expressed between male and female elongated embryos developed in cows (Fig 6B). However, compared to the number of DEmiRNAs detected in CM vs. CF, the number of DEmiRNAs identified in HM vs. HF was 8 folds lower. In silico functional analysis of the DEmiRNAs identified between male and female embryos developed in heifers indicated that endocytosis, immune systems related pathways, cell cycle and AMPK signalling pathways were enriched by the target genes of these miRNAs (Fig 8B).

### The miRNA expression profile differences between the male or female embryos of cows and heifers

**Fig 9:**
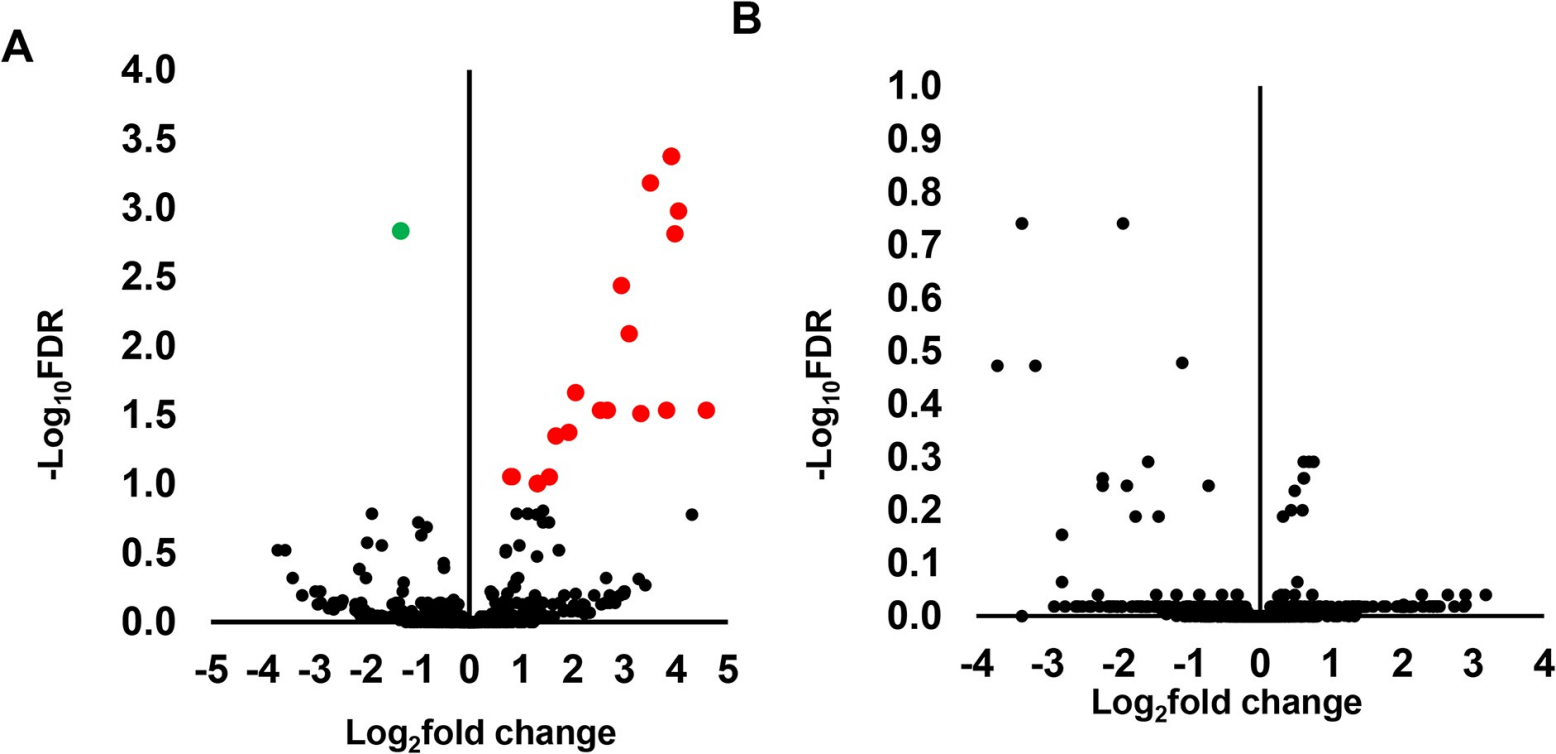
Volcano plot showing the average (log_2_ fold change) expression of miRNAs in CM compared to HM (A) and in CF compared to HF (B). Red and green dots represent the up and downregulated miRNAs, respectively in CM compared to the HM group. Black dots represent miRNAs that did not show significant expression differences between groups.

**Fig 10:**
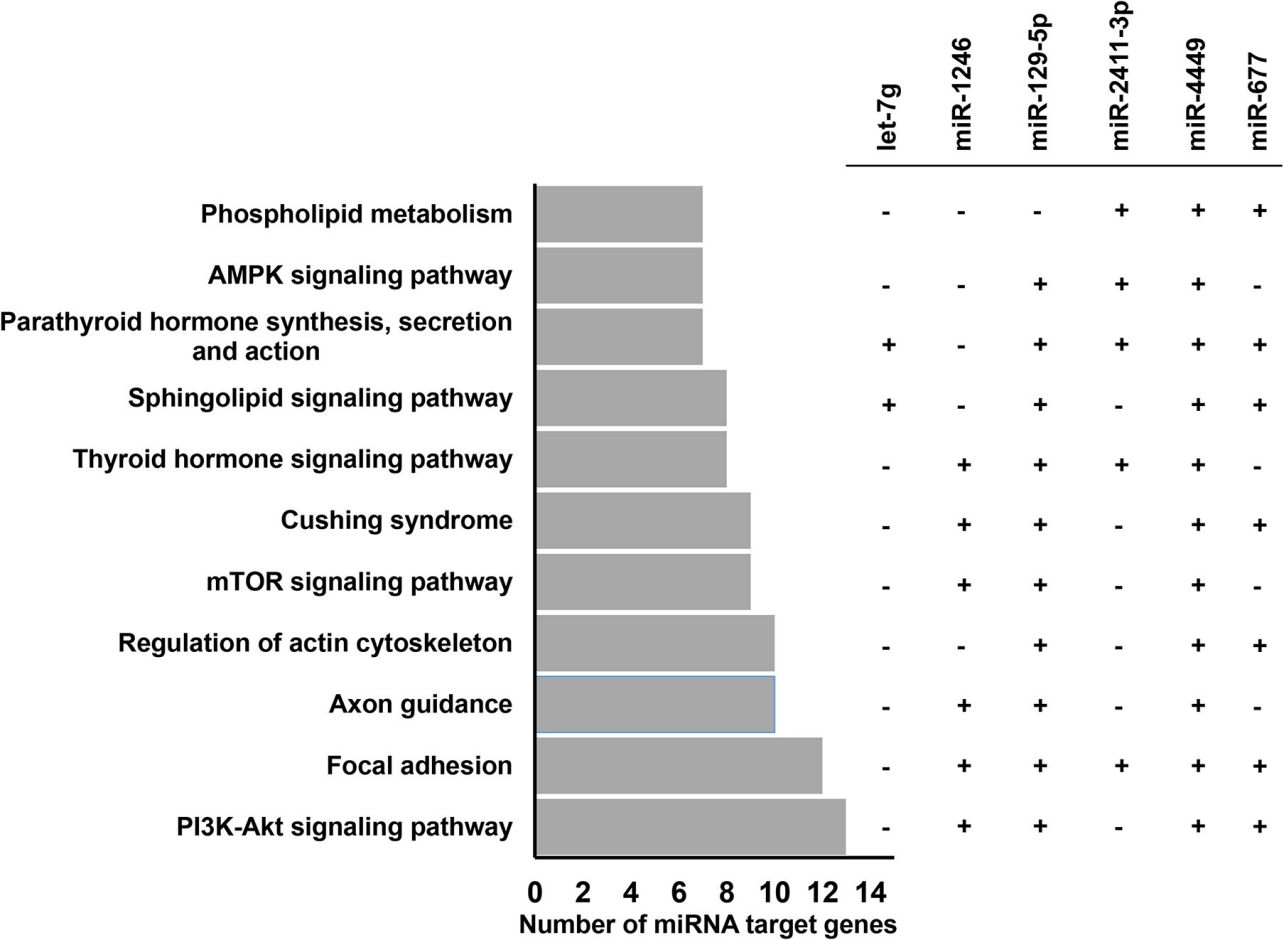
Molecular pathways enriched by differentially expressed miRNAs between CM and HM. The plus (+) and minus (-) symbols indicate the presence and absence of miRNAs in the specific pathway, respectively.

To investigate the extent of posttranscriptional regulation responses of the male embryos to the maternal environment on day 13 of the gestation period, the miRNA expression profile of the CM was compared with the HM ones. Similarly, to understand, the extent of posttranscriptional regulation responses of the female embryos with the maternal environment, the miRNA expression profile of the CF was compared with that of the HF ones. While 19 miRNAs were significantly differentially expressed between CM and HM (Fig 9A), there were no significantly altered miRNAs between the CF and HF group (Fig 9B). Among miRNAs altered between CM and HM, the expression levels of 12 miRNAs including bta-miR-12023, bta-miR-11985, bta-miR-11976 and bta-miR-2411-3p were increased with > 4 folds in male embryos developed in cows compared to those developed in heifers (Table 3). The significant relevance of these DEmiRNAs with respect to embryo elongation and adaptation to the maternal environment was studied by analyzing the pathways enriched by target genes of these miRNAs. These DEmiRNAs were involved in PI3K-Akt signaling pathway, focal adhesion, axon guidance, cellular energy homeostasis pathways (mTOR signaling pathway & AMPK signalling pathway), phospholipid metabolism, parathyroid hormone synthesis, secretion and action and thyroid hormone signaling pathways (Fig 10, S6 Table).

**Table 3:** Top differentially expressed miRNAs between the CM and HM embryo groups.

miRNA	Log_2_FC	P value	FDR
bta-miR-12023	4.6	0.0004	0.0292
bta-miR-11985	4.0	0.0000	0.0011
bta-miR-11976	3.9	0.0000	0.0004
bta-miR-11975	3.9	0.0000	0.0004
bta-miR-11972	3.8	0.0003	0.0292
bta-miR-1949	3.5	0.0000	0.0007
bta-miR-11987	3.3	0.0004	0.0309
bta-miR-2427	3.1	0.0001	0.0081
bta-miR-2411-3p	2.9	0.0000	0.0037
bta-miR-4449	2.7	0.0003	0.0292
bta-miR-2332	2.5	0.0004	0.0292
bta-miR-1246	2.1	0.0002	0.0218

FDR: False discovery rate

### Sexual dimorphic miRNA expression in embryos irrespective of the maternal environment

**Fig 11:**
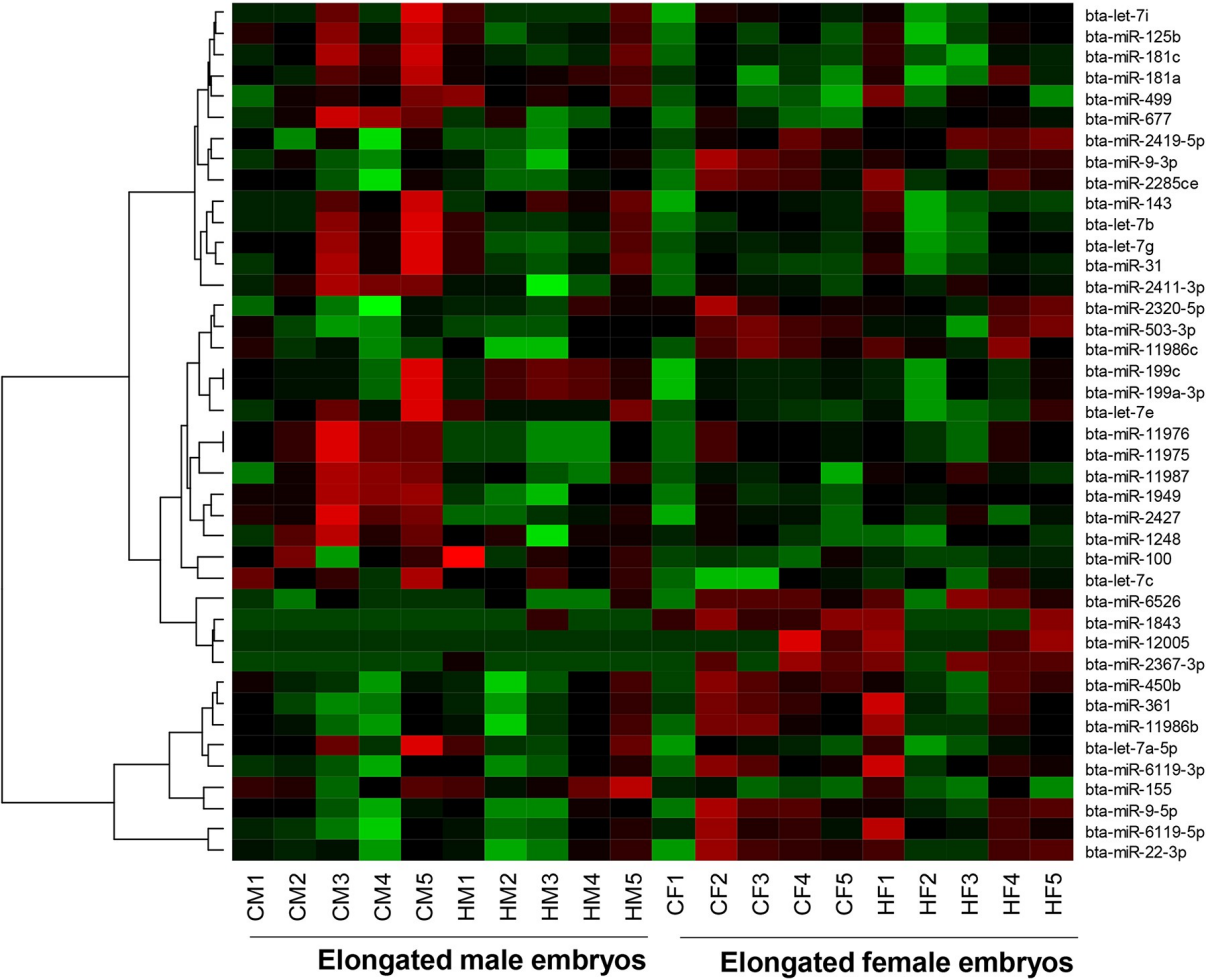
Heatmap depicting the expression pattern of differentially expressed miRNAs between the male and female embryos irrespective of the reproductive microenvironment of the maternal environment. Red and green colours indicate the up and downregulated miRNAs in elongated male embryos compared to female counterparts.

With the aim to investigate sexual dimorphic miRNA expression in the male and female embryo at the elongation stage, irrespective of the effect of the maternal environment, we performed a differential expression analysis between the male and female embryos by omitting the second factor, i.e. the recipients. For this, the miRNA expression data of the male embryos of cows and heifers were pooled in one group and the female embryos from cows and heifers were pooled in the second group. Differential expression analysis indicated a significant upregulation of 24 miRNA including the let-7 families (bta-let-7a-5p, bta-let-7b, bta-let-7c, bta-let-7e, bta-let-7g and bta-let-7i) and downregulation of 12 miRNAs including bta-miR-9-5p, bta-miR-9-3p, bta-miR-6119-3p and bta-miR-450b in male elongated embryos compared to the female ones (Fig 11).

**Fig 12:**
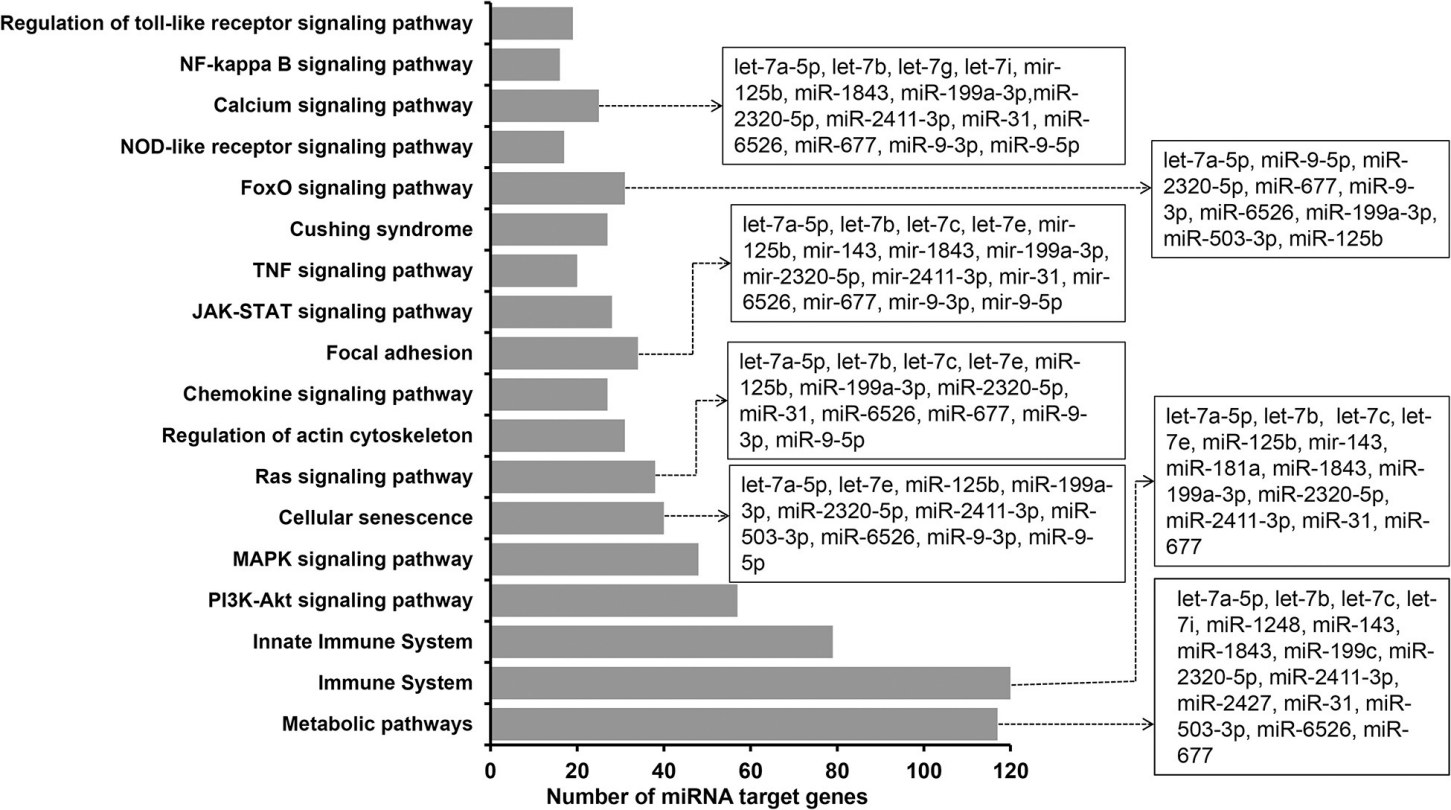
Molecular pathways enriched by the differentially expressed miRNAs between elongated male and female embryos.

The functional relevance of the sexually dimorphic differentially expressed miRNAs with respect to embryonic survival and adaptability was investigated using their target genes. Of the differentially expressed miRNAs between the male and female elongated embryos, 32 miRNAs including mir-9-3p and the let-7 families (let-7a-5p, -7b, -7c, -7e, -7g and let-7i) were found to potentially target 1566 genes. Of these, the miRNet identified 70 target genes including *AGO1*, 63 genes including *XIAP*, *and GATA6*, 50 genes including *YY1 and*, 31 target genes including *AGO4* and *DICER1*, as validated target genes of the miR-9-5p, miR-181a-5p, miR-31-5p and let-7a-5p, respectively. These miRNAs were involved in key molecular pathways including metabolic pathways, cellular censuses, focal adhesion, ras signaling, immune related pathways, calcium signaling and foxO signaling (Fig 12).

## Discussion

Sex specific embryo survival involving sex specific coping mechanisms against the developmental insults, including the suboptimal physiological condition of the mother, fosters the pregnancy success of one sex than the other. In the current study, the cows seem to favour male embryo survival while the heifer’s environment was not biased towards the male or the female male embryos (Table 1). The skewed sex ratio in cows, but relatively the absence of this phenomenon in heifers may explain the sex biased embryo loss or survival according to the physiological status of the recipients. Thus, sex specific survival of embryos under different physiological conditions could be associated with their ability to establish bidirectional communication with the maternal environment by secreting sex specific molecules. The sex specific molecules such as miRNAs may help the embryo to signal its presence in its maternal environment by regulating the expression of various genes involved in a cell-to-cell communication and signaling pathways involved in developmental processes.

### MicroRNAs that possibly involved in embryo elongation on day 13 of the gestation period

Since a single miRNA can target several genes in a given pathway, focusing on the sex specific role of miRNAs during embryo elongation will provide greater opportunities for developing sex specific embryonic miRNA markers associated with development and survival. Therefore, in the current study, we were interested in identifying miRNAs that are expressed in female and male elongated embryos developed in cows or nulliparous heifers. Accordingly, at least 254 miRNAs were detected in all male and female elongating embryo samples (Fig 2B, S1 Table). This may in turn suggest that these miRNAs may have a housekeeping role during embryo elongation independent of the sex of the embryo and the environment in which they developed. However, further research is required to determine the potential role of those miRNAs in various physiological processes of embryo elongation and implantation. Nevertheless, in the current study, the involvement of highly expressed miRNAs in pathways related to immune response, cell proliferation and elongation, and cell-to-cell communication suggests that these miRNAs could be directly or indirectly required for embryo elongation and implantation by regulating various genes involved in various developmental processes. For instance, miR-10b, miR-148a, miR-21-5p, miR-378, miR-22-3p, and miR-10a and miR-30d were commonly involved in cell cycle and endocytosis. The cell cycle is critical during embryonic development for regulating the pace of cell proliferation and timing of cell cycle progression [43], whereas endocytosis during development may be required for gastrulation [44, 45]. But to what extent, these miRNAs regulate embryonic development via these pathways remains the future research interest. Nevertheless, previous studies demonstrated the relevance of some of these miRNAs in embryogenesis. For instance, increased expression of miR-10a and miR-10b during embryonic development could be required for proper angiogenesis which is one aspect of proper embryonic development [46] and miR-21-5p may be required for embryo implantation [47] and it was implicated as a potential biomarker for pregnancy establishment [48]. Likewise, overexpression of miR-30d could increase embryo adhesion to the maternal environment [49], and improve implantation rate and fetal development by increasing maternal receptivity [50]. Similarly, miR-148a and miR-378 were reported to be associated with enhanced early embryonic development [51] and blastocyst hatching [52], respectively.

In addition, miR-26a was one of the top miRNAs enriched in male and female elongated embryos developed in cows or heifers. Gene ontology analysis has shown that this miRNA along with miR-21-5p, miR-22-3p and miR-10a is involved in the Hippo signaling pathway, which is essential for trophoblastic differentiation and inner cell mass formation [53]. The available data showed that miR-26a can stimulate trophoblastic proliferation [54] and can be a potential biomarker of early pregnancy [55]. Taken together, miRNAs enriched in both male and female embryos are found to be involved in various key processes that are important for the development and survival of elongated bovine embryos for subsequent establishment and maintenance of pregnancy.

### Sexual dimorphic miRNA expression patterns associated with adaptation to suboptimal maternal environment

Embryo losses in cows are more frequent compared to heifers [9], partly due to factors associated with negative energy balance [8]. Thus, we attempted to investigate the molecular response of the male and the female embryos towards the cow’s and the heifer’s maternal reproductive microenvironment by analyzing DEmiRNAs between the male and female embryos developed in cows as well as in heifers. Interestingly, the number of DEmiRNAs detected between the male and female embryos in cows was 8 times higher compared to the DEmiRNAs detected between male and female embryos developed in heifers (Figs 6 and 8) suggesting that the reproductive microenvironment of the cows may foster the sexual dimorphic miRNA expression as a response to the maternal environment. Here, the main question that needs to be answered is what triggers the increased number of DEmiRNAs between the male and female embryos that were developed in cows, but not in heifers? Although its fate after day 13 of the gestation period is not known, the developmental data from the current study showed that compared to the female ones, the male embryos were able to adapt to the cow’s microenvironment until day 13 of the gestation period. The oviduct and uterine microenvironment in cows could be affected by increased metabolic stress such as non-esterified volatile fatty acids and beta hydroxybutyrate at the early stage of lactation [56]. Thus, embryos that can survive in such a microenvironment may exhibit remarkable plasticity by modifying signaling pathways including those associated with the immune system. With this respect, when we looked into the functional annotations of DEmiRNAs identified between male and female embryos that were developed in cows, 14 differentially expressed miRNAs including let-7a-5p and let-7e were involved immune related pathways including the innate immune system, TNF signaling, NF-kappa B signaling and chemokine signaling (Fig 7, S5 Table). For instance, the role of let-7 family miRNA in regulating the innate immune response in facilitating robust developmental programs even under stress conditions has been documented [57]. Immune processes are the critical factors in determining the fate of pregnancy and miRNA mediated immune system regulation has been reported in various circumstances [58, 59]. Thus, it is possible to suggest that the male embryos can adapt to the maternal environment by regulating transcripts that are associated with the immune system, although further research is required to reach a valid conclusion.

In addition to the immune system, the mammalian target of the rapamycin (mTOR) signaling pathway was also among the key pathways enriched by differentially expressed miRNAs between the male and female elongating embryos developed in cows. Nine DEmiRNAs including let-7a-5p, let-7b, let-7c, let-7d, let-7e, miR-225b, and miR-555 were involved in mTOR pathway (Fig 7, S5 Table). Of these, let-7a-5p is believed to regulate the mTOR signaling by regulating the expression of *MAP4K3*, a gene which is responsible for the formation of mTORC1 in the mTOR signaling cascade [60]. Similarly, miR-125b is involved in mTOR signaling pathway by targeting *PIK3CD* and regulate cell proliferation and cell cycle progression [61] and miR-155 involves in autophagy processes by targeting *RHEB*, *RICTOR* and *RPS6KB2* genes which are essential in the mTOR pathway [62]. It is believed that embryo elongation involves the exponential increases in length and weight of the trophectoderm which supports uterine epithelial-derived histotroph [63]. Nevertheless, to what extent does the mTOR signaling regulate embryo survival in suboptimal development environments? mTOR signaling, which is evolutionarily conserved serine/threonine kinase, involves in regulating many biological processes including cell growth, proliferation and survival, and metabolism, by sensing and integrating intracellular and extracellular signals in the form mTOR complex 1 (mTORC1) and mTOR complex 2 (mTORC2) [64, 65] and it is a key signaling pathway in regulating cellular homeostasis in an environmental stressor-dependent manner [66]. Inactivation of the mTOR signaling can impair cell proliferation in both embryonic and extraembryonic compartments followed by the death of the embryo soon after implantation [67]. The mTOR signaling also regulates embryonic diapause, the temporary suspension of development of the embryo occurs due to the consequence of adverse environmental conditions or metabolic stress, when further embryo development could be risky [68]. Therefore, regulating the mTOR signaling pathway could be one of the key processes that can be triggered by embryos in a sex specific manner for its further development, adaptation and survival in suboptimal developmental conditions.

### Male elongated embryo is more susceptible to altering its miRNA expression in response to maternal environments

Once we confirmed the presence of miRNA expression divergences between the male and female elongated embryos developed in cows than heifers, we also opted to obtain an additional insight into the effect of maternal environment and the sex specific response of the embryos by comparing the miRNA expression profile of the male elongated embryos of the cows with the male elongated embryos of the heifers, and the female elongated embryos of the cows with the female elongated embryos of the heifers. Interestingly, no significantly differential expression of miRNAs was detected between the female embryos developed in cows and heifers, but the expression level of 18 miRNAs was increased in the male elongated embryos developed in cows compared to the male elongated embryos developed in heifers (Fig 9). Therefore, this data provided clear information that the maternal environment has more impact on the male embryo miRNA expression compared to the female ones. This result seems to be coinciding with the developmental data by which the sex ratio of the elongated embryos that were developed in cows was skewed to males (Table 1). This in turn reveals the increased survival rate of the male embryos than females in the cow’s microenvironment. Previous studies on mRNA expression also indicated that suboptimal culture conditions exhibited a 3 fold increase in the number of differentially expressed genes compared to their female counterparts [26]. Moreover, while comparing the in vitro produced male and female embryos to their in vivo counterparts at day 32 of the gestation period, [69] also indicated about 12 times more differentially expressed genes between males were detected compared to those detected between females. This may indicate that male embryos are more susceptible to alterations in gene expression due to suboptimal developmental conditions. Therefore, 8 fold increase in the number of DEmiRNAs between the male elongated embryos compared to the number of DEmiRNAs detected between the female elongated embryos. This may suggest the that the male embryos could respond to suboptimal maternal environment by modulating their gene regulatory machinery for its survival and maintain pregnancy.

**Fig 13:**
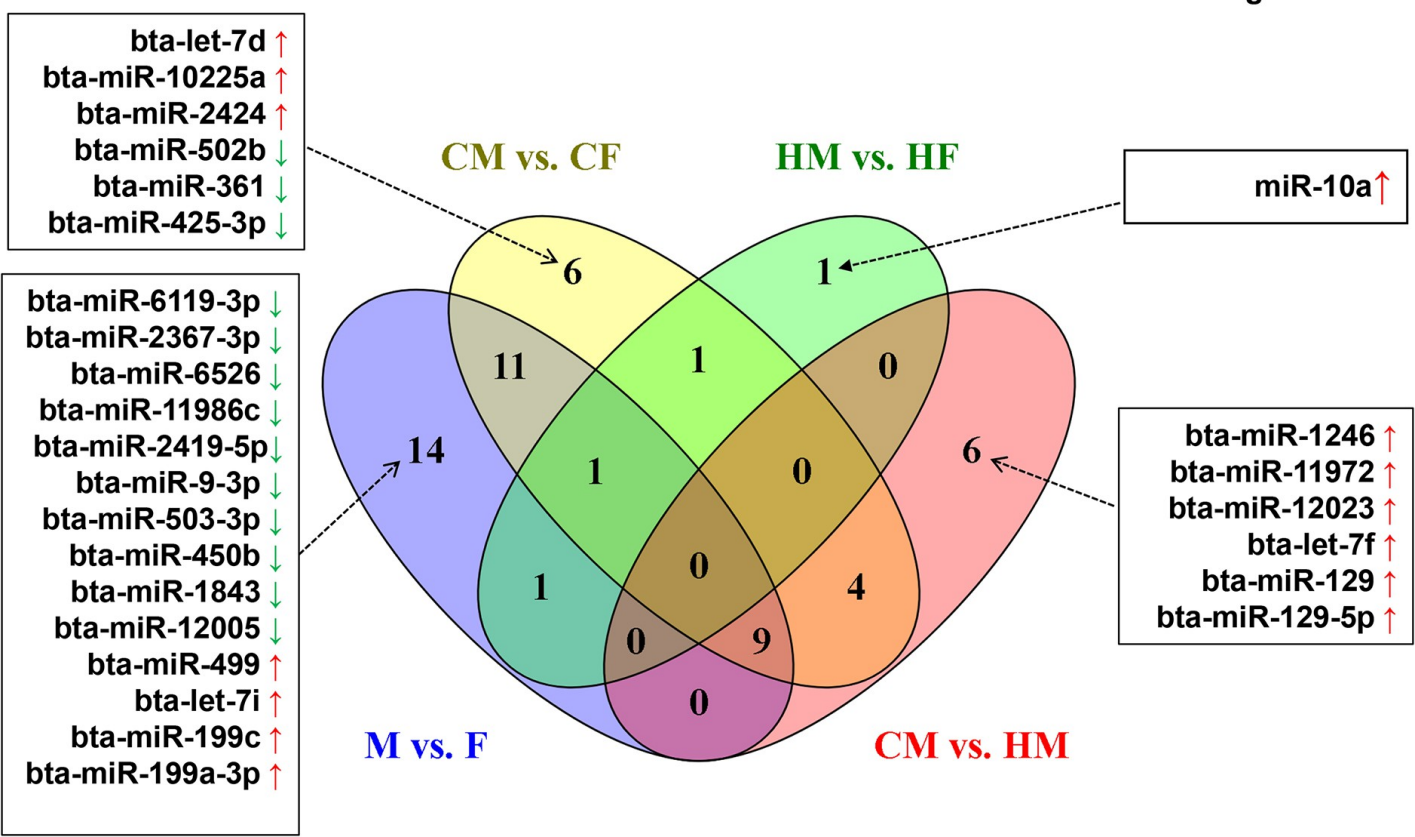
Venn diagram depicting exclusively and commonly expressed miRNAs in CM vs. CF, HM vs. HF, and CM vs. HM and M vs. F. Since, no significantly differentially expressed miRNAs were detected in CF vs. HF, this comparison was not included in the Venn diagram. Arrows ↑ and ↓ indicated the upregulation and downregulation of miRNAs, respectively in the first compared to the latter group.

It is also interesting to identify individual miRNA that are solely associated with sexual dimorphism and/or the maternal environment. Perhaps, merging the DEmiRNAs obtained from different comparisons (CM vs. CF, HM vs. HF, CM vs. HM and M vs. F) using the Venn diagram (Fig 13) and looking into exclusive and common DEmiRNAs may help to filter miRNAs that are specific to the intrinsic characteristics of the embryos (sexual dimorphism) or altered by the maternal environment. Accordingly, it appears that at least four miRNA expression patterns, namely sex biased miRNAs expression patterns which are directly associated with the sex of embryos, maternal environment induced sex biased expression patterns, maternal environment induced expression patterns only in the male embryos and expression patterns that can be altered by the confounding effect of maternal environment and sex of embryos can be identified. For instance, it appears that 14 miRNAs including bta-miR-9-3p, bta-miR-499, bta- miR-199a-3p, bta-miR-6526, bta-miR-11986c, bta-miR-503-3p and, bta-miR-450b were differentially expressed between male (M) and female (F) embryos when the maternal environment was omitted from analysis suggesting that these miRNAs may be mainly affected the sex of the embryo than any other factors. The majority of these were downregulated in males but increased in female embryos. For instance, bta-miR-6119-3p, bta-miR-6526 and bta-miR-11986c which were significantly decreased in male embryos are transcribed on X chromosome and others including bta-miR-9-3p, bta-miR-499 are transcribed from autosomal chromosomes. Although further research is required to track the function of these miRNAs in male and female embryogenesis, it is speculated that these miRNAs could be associated with various sex specific processes of embryo development including sex differentiation and gonadal development. Previous reports have also indicated the role of miR-199-3p in granulosa cell migration and steroid genesis in goose ovarian follicles [70], sex biased expression of miR-9-3p on day 15.5 of mouse development [71] and in 1–3 old yellow catfish [72] and miR-499 in the gonads of 1–4 years of Chinese giant salamander [73]. On the other hand, 6 DEmiRNAs (bta-let-7d, bta-miR-10225a, bta-miR-2424, bta-miR-502b, bta-miR-361, bta-miR-425-3p) were specific to the CM vs. CF whereas bta-miR-10a was specific to the HM vs. HF comparison suggesting that the sex biased expression patterns of these miRNAs at day 13 of the gestation period might be mainly induced by the maternal environment. Thus, these miRNAs can be considered as maternal environment induced sex-biased miRNAs. Similarly, altered expression of bta-miR-1246, bta-miR-11972, bta-miR-12023, bta-let-7f, bta-miR-129 and bta-miR-129-5p between the CM and HM may indicate that the expression patterns of these miRNAs can be altered exclusively in male embryos in response to the maternal environment. For instance, miR-1246 was highly expressed in the serum of heat-stressed Holstein cows [74] and a significant increase in primary human trophoblast cells cultured in 20% compared to 10% O_2_ concentration [75] may indicate that the expression profile of miR-1246 can be altered by stress condition. Moreover, this miRNA was found to be significantly increased on day 21 of trophectoderm cells cultured in vitro suggesting the involvement of this miRNA in the maternal-foetal cross-talk [76].

## Conclusion

The current study revealed the outline of the molecular responses of male and female embryos to maternal microenvironments by modifying their miRNA abundances, which is required for fine-tuning the activity of genes required for the development and survival. Interestingly, sexual dimorphic differences in miRNA expression have been manifested when embryos developed in the cows, which are supposed to be suboptimal environments, compared to their heifer counterparts. Especially male elongated embryos tend to respond to the unfavorable maternal microenvironment better than the female which might be associated with intrinsic sexual dimorphic ability to modulate the miRNA machinery. This has been further supported by that those candidate DE miRNAs between male and female embryos developed in cows were involved in various key processes including immune system regulation, mTOR signalling, cell-to-cell communication, cell proliferation and differentiation signalling pathways. Furthermore, irrespective of the origin of the embryos, 36 miRNAs that were involved in various pathways including metabolic pathways, immune related pathways, cell division and communication were differentially expressed in male and female elongated embryos. This may in turn provide a clear picture of the sexual dimorphic miRNA dynamics during the early elongation period. Overall, this study indicated that the increased sex ratio towards males in embryos developed in cows was also complemented by increased divergences in sexual dimorphic miRNA expression between male and female embryos as a response to the maternal microenvironment. The male elongated embryos were found to respond to suboptimal maternal condition by modifying their miRNA expression which might be crucial for their survival as evidenced in the skewed sex ratio of elongated embryos developed in the cow’s environment.

## Supporting information

S1 Table miRNAs detected in all biological replicates of CM, CF, HM and HF. (XLSX)

S2 Table Highly expressed miRNAs and their target gene interaction. (XLSX)

S3 Table Validated targets of the miR-22-3p and miR-21-5p. (XLSX)

S4 Table Differentially expressed miRNAs between CM and CF and their validated target genes obtained using miRNet analysis tool. (XLSX)

S5 Table Differentially expressed miRNAs between CM and CF groups and the pathways enriched by their target genes. (XLSX)

S6 Table Differentially expressed miRNAs between CM and HM groups and the pathways enriched by their target genes. (XLSX)
